# *Xanthophyllomyces dendrorhous*-Derived Astaxanthin Regulates Lipid Metabolism and Gut Microbiota in Obese Mice Induced by A High-Fat Diet

**DOI:** 10.3390/md17060337

**Published:** 2019-06-05

**Authors:** Jihui Wang, Shiwen Liu, Han Wang, Shan Xiao, Cheng Li, Ying Li, Bingnan Liu

**Affiliations:** 1 Engineering Research Center of Health Food Design & Nutrition Regulation, School of Chemical Engineering and Energy Technology, Dongguan University of Technology, Dongguan 523808, China; wangjh_dlpu@163.com (J.W.); ykslsw@163.com (S.L.); xiaoshan@dlpu.edu.cn (S.X.); 2 School of Biological Engineering, Dalian Polytechnic University, Dalian 116034, China; hwang@dlpu.edu.cn (H.W.); licheng19850724@126.com (C.L.); 3 School of Food Science and Engineering, Dalian Ocean University, Dalian 116023, China; liying@dlou.edu.cn

**Keywords:** astaxanthin, *Xanthophyllomyces dendrorhous*, lipid metabolism, gut microbiota, obesity

## Abstract

Astaxanthin is an important antioxidant with many biological activities such as anti-tumor, anti-obesity, cardioprotective, and immuno-modulatory activities. Most of these biological activities are derived from (3S,3′S)-astaxanthin, while the activities of (3R,3′R)-astaxanthin are rarely reported. The purpose of this study was to investigate the effect of (3R,3′R)-astaxanthin on lipid metabolism and gut microbiota in mice fed with a high-fat diet. In this work, 40 male C57BL/6 mice were divided into 8 groups fed a high-fat diet supplemented or not with (3R,3′R)-astaxanthin or *Xanthophyllomyces dendrorhous* for 8 weeks. The weight gain, energy intake, fat index, plasma triacylglycerol and cholesterol, liver triacylglycerol and cholesterol, and gut microbiota were determined. The results showed that the addition of (3R,3′R)-astaxanthin/*X. dendrorhous* to the high-fat diet as a supplement prevented weight gain, reduced plasma and liver triacylglycerol, and decreased plasma and liver total cholesterol. The addition of (3R,3′R)-astaxanthin/*X. dendrorhous* also regulated the gut microbiota of the mice, which optimized the ratio of *Bacteroides* to *Firmicutes* and increased the content of *Verrucomicrobia*, especially *Akkermansia*. The changes in the gut microflora achieved a healthier structure, thus reducing the incidence of obesity. Thus (3R,3′R)-Astaxanthin has the function of regulating lipid metabolism and gut microbiota to prevent obesity caused by a high-fat diet. The production strain of (3R,3′R)-astaxanthin, *X. dendrorhous*, has the same function as astaxanthin in preventing obesity caused by a high-fat diet, which reflects its potential ability as a probiotic drug.

## 1. Introduction

Astaxanthin is an oxidized derivative of carotenoids. It mainly occurs in the marine environment, leading to the red color of seafood such as shrimp, crab, and salmon. However, animals cannot produce astaxanthin de novo; they can only accumulate astaxanthin in the body by ingesting microorganisms such as *Haematococcus pluvialis* and *Xanthophyllomyces dendrorhous* in the environment [1]. The prominent property of astaxanthin is its strong antioxidant capacity, which can effectively improve the body’s immune system [2,3]. Astaxanthin has great potential in the treatment and prevention of cardiovascular diseases, tumors, and certain immune system diseases [4,5,6]. 

Astaxanthin has three stereoisomers: (3S,3′S), (3R,3′R), and (3R,3′S). Chemically synthesized astaxanthin is a racemic-mixture of the three isomers [1]. Compared with chemically synthesized astaxanthin, natural astaxanthin shows better activity. For commercial astaxanthin production, natural astaxanthin is currently mostly derived from *H. pluvialis* and *X. dendrorhous*, which produce (3S,3′S)- and (3R,3′R)-astaxanthin, respectively [7,8]. Moreover, astaxanthin from *H. pluvialis* exists as free, mono, and di-esters, while in *X. dendrorhous,* it is present in a free form that is more easily utilized [9]. However, astaxanthin available as a nutraceutical or drug on the market is currently dominated by products derived from *H. pluvialis* because most research regarding the biological activity is related to *H. pluvialis*-derived astaxanthin. For example, compared with other carotenoids, astaxanthin showed the greatest antioxidant activity [10]. For (3R,3′R)-astaxanthin, *X. dendrorhous* is one of the few natural sources and is a very promising candidate for commercial production, as it is well accessible by bioprocess engineering and costs less [11]. It is therefore conceivable that the biological activities of *X. dendrorhous*-derived astaxanthin will expand the application of astaxanthin, especially for medical purposes.

Obesity is a common metabolic syndrome. When the human body takes in more calories than it consumes, the excess calories are stored in the body in the form of fat, the amount of which exceeds the normal physiological demand and evolves into obesity. Obesity is on the rise in most parts of the world, and it causes many other diseases, including hypertension, hyperlipidemia, and diabetes [12,13,14]. Therefore, preventing obesity is important for a healthy life. An important cause of obesity is a high-fat diet, which is common today. Whether astaxanthin can be used as an anti-obesity drug is not known, and its effect on obesity caused by high-fat diet is worth studying. A previous study indicated an obesity-reducing effect of esterified (3S,3′S)-astaxanthin from *H. pluvialis* [15]. Whether the free (3R,3′R)-astaxanthin from *X. dendrorhous* has lipid-lowering function needs further study.

In this study, the free (3R,3′R)-astaxanthin derived from *X. dendrorhous* was added to the feed to verify its impact on body weight, lipid metabolism, and gut microflora. Separately, *X. dendrorhous* powder was added to the feed in the control group to examine the difference in activity between crude and pure astaxanthin products. The aim of this study was to investigate the effects of *X. dendrorhous*-derived astaxantithin on obese mice.

## 2. Results

### 2.1. Effects of X. dendrorhous-Derived Astaxanthin on Lipid Metabolism and Gut Microbiota

#### 2.1.1. Body Weight and Lipid Content

Astaxanthin from *X. dendrorhous* was added to the high-fat diet (HFD), and the mice were weighed weekly. After 8 weeks, no significant difference in tail length was observed among the groups. The weight gain of mice in each group is shown in Table 1. With the increase in feeding time, the body weight of the mice showed an increasing trend, and compared with the other groups, the HFD group gained weight the fastest. From the second week of feeding, the weight gain of the HFD_2ASX (high-fat diet + astaxanthin (0.01%)) group was significantly lower than that of the HFD group (*p* < 0.01). However, no significant difference was observed between the HFD_ASX (high-fat diet + astaxanthin (0.005%)) group and the HFD group. During the feeding period, the food intake of each group of mice was recorded every week. After the feeding, the average energy intake of each group of mice was calculated. The results are shown in Table 1. No significant difference in energy intake was observed among the HFD, HFD_ASX, and HFD_2ASX groups, indicating that the change in body weight was affected by the addition of astaxanthin in the feed rather than the energy intake. The results showed that astaxanthin from *X. dendrorhous* reduced the body weight gain induced by the high-fat diet, and the effect was dose-dependent. The fat index showed a correlation with body weight. The body fat index of the mice in the HFD_ASX group and the HFD_2ASX group was significantly lower than that in the HFD group (*p* < 0.01), indicating that astaxanthin derived from *X. dendrorhous* reduced the body fat content.

The triglyceride and total cholesterol levels in the plasma and liver are shown in Table 1. Compared with the HFD group, the content of triglyceride and total cholesterol in the HFD_2ASX group was significantly lower (*p* < 0.05) in either the plasma or liver. The results showed that astaxanthin from *X. dendrorhous* reduced the content of triglyceride and total cholesterol in the plasma.

#### 2.1.2. Gut Microbiota

**Figure 1:**
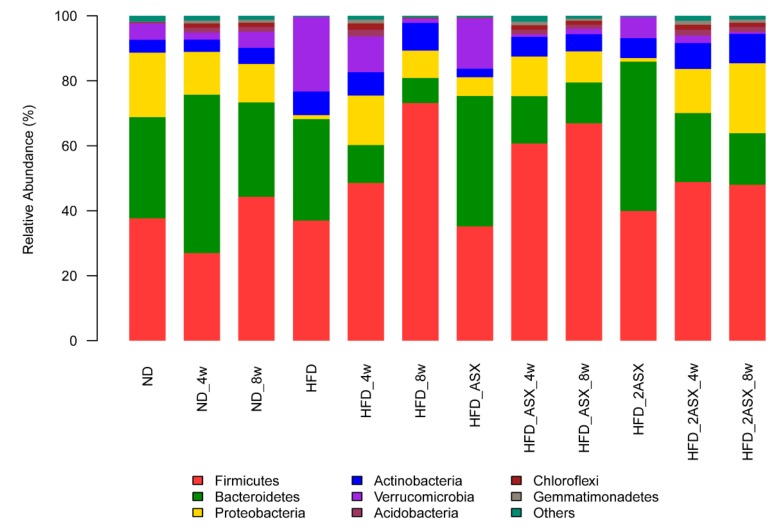
Effects of *X. dendrorhous*-derived astaxanthin on gut microbiota at the phylum level in the mice fed with a high-fat diet. HFD, HFD_4w, and HFD_8w: fed with the high-fat diet for 0, 4, 8 weeks, respectively; HFD_ASX, HFD_ASX_4w, and HFD_ASX_8w: fed with the high-fat diet + astaxanthin (0.005%) for 0, 4, 8 weeks, respectively; HFD_2ASX, HFD_2ASX_4w, and HFD_2ASX _8w: fed with the high-fat diet + astaxanthin (0.01%) for 0, 4, 8 weeks, respectively; ND, ND_4w, and ND_8w: fed with the normal diet for 0, 4, 8 weeks, respectively.

Four-week-old SPF C57BL/6J mice were fed with the standard feed for one week. The feces of the mice were collected, and the gut microbiota was analyzed at the beginning, the fourth week, and the eighth week of the experiment (Figure 1). In this paper, several microflora phyla involved in body weight regulation were analyzed.

*Bacteroidetes* is usually found in the gut of humans or animals. In the HFD group, the *Bacteroidetes* content decreased by 23.51%, from 31.22% to 7.71%. In the HFD_ASX group and HFD_2ASX group, the *Bacteroidetes* content decreased from 40.22% to 12.58%, and 46.01% to 15.89%, respectively. *Firmicutes* is a large group of bacteria, which is usually analyzed simultaneously with *Bacteroidetes*, because the ratio between them affects the body's absorption of calories in food and plays a role in weight gain or loss. When there are more *Firmicutes* than *Bacteroidetes* in the gut, the body will increase the absorption of calories in food, leading to obesity. As shown in Figure 1, in the HFD group, the content of *Firmicutes* increased from 37.01% to 73.19%; the content of *Firmicutes* in the HFD_ASX group and HFD_2ASX group increased from 35.14% to 66.91% and 39.93% to 47.95%, respectively. The ratio of *Firmicutes* to *Bacteroidetes* was increased from 1.19, 0.87, and 0.87 to 9.49, 3.17, and 3.02 in the HFD, HFD_ASX, and HFD_2ASX groups, respectively. The high-fat diet significantly increased the ratio of *Firmicutes* to *Bacteroidetes*, but astaxanthin from *X. dendrorhous* slowed the trend.

*Verrucomicrobia* is found mainly in aquatic and soil environments, as well as in human feces. Studies have shown that the content of *Akkermansia* (Akk) in *Verrucomicrobia* is 83% [16]. Akk is currently known as ”lean bacteria”. As a probiotic, it has attracted extensive attention in recent years [17]. As shown in Figure 1, in the HFD group, the content of *Verrucomicrobia* decreased by 21.68%, and it decreased by 13.8% and 6.03% in the HFD_ASX and HFD_2ASX groups, respectively. This result showed that high-fat diet consumption led to a significant decrease in the content of *Verrucomicrobia* in the gut microbiota, while the addition of astaxanthin in the high-fat diet could control the reduction of *Verrucomicrobia*, and this effect was dose-dependent.

The gut microbiota showed similar results at the species level as at the phylum level (Supplementary Figure S1). The results also showed that consumption of the high-fat diet led to a significant decrease in the content of Akk in the gut microbiota, while the addition of astaxanthin in the high-fat feed could control the decrease in the content of Akk. This indicated that astaxanthin had a beneficial effect on Akk as a prebiotic and could increase the abundance of Akk in the gut microbiota.

### 2.2. Effects of X. Dendrorhous on Lipid Metabolism and Gut Microbiota

#### 2.2.1. Body Weight and Lipid Content

On the premise that there was no significant difference in energy intake of the mice among the HFD’-fed groups, the body weight of the HFD’ group increased the fastest (Table 2). A significant difference in body weight between the HFD’_2XD (high-fat diet + *X. dendrorhous* (20% *w*/*w*)) group and the HFD’ group (*p* < 0.05) was observed, while no significant difference was observed in body weight between the HFD’_XD group (high-fat diet + *X. dendrorhous* (10% *w*/*w*)) and the HFD’ group. The fat index of the HFD’_XD group and HFD’_2XD group was significantly lower than that of the HFD’ group (*p* < 0.01).

The triglyceride and total cholesterol levels in the plasma and liver are shown in Table 2. Compared with the HFD’ group, the content of triglyceride and total cholesterol in the HFD’_XD and HFD’_2XD groups was significantly lower (*p* < 0.05). The results showed that *X. dendrorhous* reduced the contents of triglyceride and total cholesterol in the plasma and liver.

#### 2.2.2. Gut Microbiota

**Figure 2:**
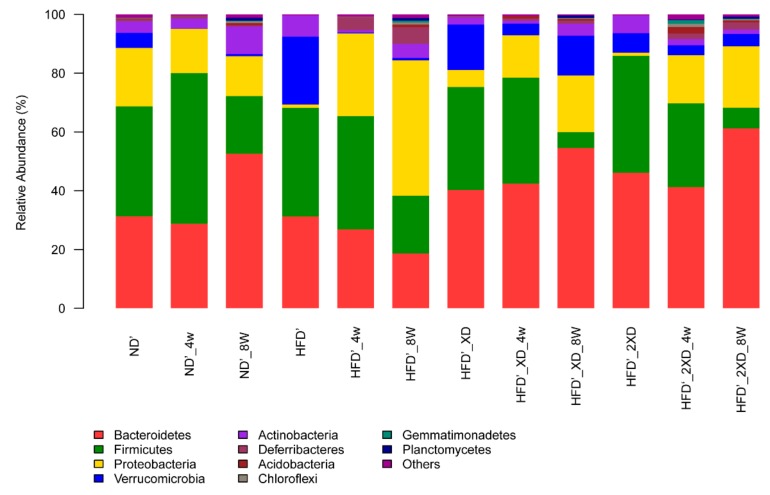
Effects of *X. dendrorhous* powder on gut microbiota at the phylum level in mice fed with a high-fat diet. HFD’, HFD’_4w, and HFD’_8w: fed with the high-fat diet for 0, 4, 8 weeks, respectively; HFD’_XD, HFD’_XD_4w, and HFD’_XD_8w: fed with the high-fat diet + *X. dendrorhous* (10% *w*/*w*) for 0, 4, 8 weeks, respectively; HFD’_2XD, HFD’_2XD_4w, and HFD’_2XD_8w: fed with the high-fat diet + *X. dendrorhous* (20% *w*/*w*) for 0, 4, 8 weeks, respectively; ND’, ND’_4w, and ND’_8w: fed with the normal diet for 0, 4, 8 weeks, respectively.

Figure 2 shows changes in the gut microbiota in each group at the phylum level. In the HFD’ group, the *Bacteroidetes* content decreased by 12.53%; the *Bacteroidetes* content in the HFD’_XD group increased by 14.21%; and the content of *Bacteroidetes* in the HFD’_2XD group increased by 15.17%. Meanwhile, the *Firmicutes* content in the HFD’ group decreased by 17.26%; the *Firmicutes* content in the HFD’_XD group decreased by 29.58%; and the *Firmicutes* content in the HFD’_2XD group decreased by 32.8%. The results showed that after adding *X. dendrorhous* in the high-fat diet, the *Bacteroidetes* content significantly increased in the gut microbiota, while the *Firmicutes* content decreased significantly. Combined with the mouse weight, the results were consistent with the report that a significant decrease in *Bacteroidetes* and an increase in *Firmicutes* were observed in obese mice [18]. 

*Proteobacteria* includes many pathogenic bacteria, such as *Escherichia coli*, *Salmonella enterica*, *Vibrio cholerae*, and *Helicobacter pylori*. In the HFD’ group, the content of *Proteobacteria* increased by 44.75%. Meanwhile, the proportion of *Proteobacteria* in the HFD’_XD group and the HFD’_2XD group increased by 13.48% and 19.79%, respectively. The results showed that the high-fat diet could promote the growth of *Proteobacteria* in the gut, which could easily cause diseases. However, adding *X. dendrorhous* in the high-fat diet can effectively inhibit the growth rate of *Proteobacteria* to keep the body healthy.

In the HFD’ group, the content of *Verrucomicrobia* decreased by 22.21%, from 23.02% to 0.81%; the content of *Verrucomicrobia* in the HFD’_XD group and the HFD’_2XD group decreased by 1.92% and 2.43%, respectively. This indicated that the consumption of a high-fat diet resulted in a significant decrease in the content of *Verrucomicrobia* in the gut microbiota, while the addition of *X. dendrorhous* in the high-fat diet maintained the content of *Verrucomicrobia*. 

The gut microbiota showed similar results at the species level as those at the phylum level (Figure S2). The content of *Bacteroides* in the HFD’ group was reduced by 10.37%; the content of *Bacteroides* in the HFD’_XD and HFD’_2XD groups increased by 12.85% and 3.81%, respectively. This showed that *X. dendrorhous* promoted the growth of *Bacteroides* and was beneficial to the health of the body. The results also showed that the addition of *X. dendrorhous* in the high-fat diet could maintain the Akk content. This indicated that *X. dendrorhous* had a beneficial effect on Akk as a prebiotic and could increase the abundance of Akk in the gut microbiota.

## 3. Discussion

Obesity not only leads to physical changes but also affects cardiovascular, respiratory, skeletal, muscle, endocrine, reproductive, and other systems and even affects social interaction, causing greater psychological stress. A high-fat diet is an important cause of obesity. However, high-fat diets such as fried foods have become a lifestyle habit of modern people around the world. The development of healthcare drugs for preventing obesity caused by high-fat diets is of great value. Esterified (3S,3′S)-astaxanthin from *H. pluvialis* had an obesity-reducing effect [15]. Whether the free (3R,3′R) -astaxanthin from *X. dendrorhous* and *X. dendrorhous* powder (crude product of astaxanthin) has lipid-lowering function needs to be investigated.

In this study, the lipid-lowering effects of astaxanthin from *X. dendrorhous* and *X. dendrorhous* powder were shown through an obese mouse model mediated by a high-fat diet. Triglycerides and total cholesterol in plasma are important indicators of obesity. The former is related to the risk of cardiovascular disease, while the latter leads to atherosclerosis. Both the free (3R,3′R)-astaxanthin from *X. dendrorhous* and *X. dendrorhous* powder had the effect of reducing triglycerides and total cholesterol in plasma. High-density lipoprotein (HDL) is mainly synthesized by the liver and small intestine. HDL can output cholesterol and promote the metabolism of cholesterol, so it plays an anti-atherosclerosis role [19]. The high dose of astaxanthin could increase the content of HDL in plasma, but the effect was not found in the *X. dendrorhous* powder (data not shown here). These results were similar to the effects of astaxanthin from *H. pluvialis* in which inhibition of the elevation in body weight and lipid appeared to be dose-dependent [15]. 

The anti-obesity mechanisms of astaxanthin may be complex. For example, astaxanthin decreased myeloperoxidase and nitric oxide synthases and made splenocytes less sensitive to lipopolysaccharide stimulation [20]; increased the usage of lipids during exercise [21]; and was a novel selective peroxisome proliferator-activated receptor gamma (PPAR-γ) modulator that acted as an antagonist or agonist to exert its ameliorative effects on obesity and insulin resistance [22]. Recently, increasing numbers of studies reported the effect of gut microbiota on obesity [23,24,25,26]. The anti-obesity property of astaxanthin might also be related to changes in gut microbiota. The results of this study revealed that astaxanthin affected the gut microbiota of the mice induced by a high-fat diet, and changes in the microbiota were correlated with changes in body weight to some extent.

The ratio of *Firmicutes* to *Bacteroidetes* is the key to body weight changes [18,27]. The ratio of *Firmicutes*/*Bacteroides* could be reduced by adding astaxanthin or *X. dendrorhous* powder to the high-fat diet. *X. dendrorhous* powder showed a stronger trend of this effect. An increased prevalence of *Proteobacteria* is a potential diagnostic signature of dysbiosis and risk of disease [28]. High-fat diets promoted the growth of *Proteobacteria* in the gut and caused diseases. However, adding astaxanthin/*X. dendrorhous* powder to the high-fat diet effectively inhibited the growth rate of *Proteobacteria*, which is beneficial to the health of the body. Akk attached to the gut mucosa, using the mucin produced by the mucosa as a source of energy, thereby protecting the gut from pathogens through competition [16]. Studies showed that feeding live Akk to mice can prevent diet-induced obesity without affecting their appetite and eating habits [29]. A high-fat diet and a large amount of alcohol will reduce the Akk content in the gut. Both astaxanthin and *X. dendrorhous* powder increase the abundance of Akk in the gut. In summary, astaxanthin and *X. dendrorhous* powder had a positive regulatory effect on gut microbiota, ensuring the health of the gut microecology and preventing obesity caused by a high-fat diet.

In this study, *X. dendrorhous* powder as a crude extract of astaxanthin was added to the high-fat diet. Since natural astaxanthin is very expensive at present, this study hoped to use crude extract to replace astaxanthin and reduce the cost without reducing its efficacy. *X. dendrorhous* powder had shown similar, and in some ways better, effects compared to natural astaxanthin in preventing obesity caused by a high-fat diet. However, its safety and other efficacy still need further study.

*X. dendrorhous*-derived astaxanthin as a liposoluble compound could bind to lipoprotein in the blood after intake [30,31]. It was inferred that free astaxanthin can be directly absorbed and utilized by the body and affect the gene expression involved in lipolysis, fatty acid oxidation, and cholesterol export. Therefore, gene expression analysis will be performed in the future to explore the mechanisms involved in the lipid reduction.

## 4. Materials and Methods 

### 4.1. Ethics Statement

All animals received humane care in accordance with the Chinese National Standard: Laboratory Animals—Guideline for ethical review of animal welfare. 

### 4.2. X. Dendrorhous and Astaxanthin

*X. dendrorhous* (CBS 6938) was purchased from Centraalbureau voor Schimmelcultures in the Netherlands. *X. dendrorhous* was fermented in potato dextrose culture medium at 20 °C using a 50 L fermenter (Guoqiang Biochemical Engineering Equipment Co., Ltd., Shanghai, China). When the concentration of astaxanthin in *X. dendrorhous* was maximized (approximately 84 h), cells were centrifuged (8000 rpm, 5 min), and then dried by a freeze dryer (Boyikang Experimental Instrument Co., Ltd., Beijing, China). The resulting characteristic red cell powder was kept at −80 °C until further use. Astaxanthin was extracted as described in the previous work [11]. Then 100 ml of *X. dendrorhous* culture broth were centrifuged at 5000× *g* for 5 min to collect cells. Pellets were mixed with 15 mL of 3 mol/L HCl (Beijing Chemical Works, Beijing, China) and incubated for 1 h. The mixture was maintained sequentially in boiling water and ice for 3 min each. Pellets were centrifuged (5000× *g*, 5 min) and washed with distilled water twice. Broken cells were extracted with 45 mL acetone (Tianjin Kemiou Chemical Reagent Co., Ltd., Tianjin, China) and centrifuged at 5000× *g* for 5 min. This process was repeated until pellets showed no red color. The extraction solution was vacuum-concentrated to 10 mL by a vacuum dryer (Taicang Hualida Laboratory Equipment Co., Ltd., Taicang, China), followed by elution with hexane:dichloromethane:acetone (5:2.5:1) mixed solvent (Tianjin Kemiou Chemical Reagent Co., Ltd., Tianjin, China). A 4 cm × 40 cm column with 80 g gum was used. The flow rate was 5 mL/min, and the red eluent was collected. Astaxanthin was purified from the extract after vacuum -drying.

### 4.3. Animals and Diet

Animals: Forty 4-week-old male SPF C57BL/J mice were purchased from Dalian Medical University (Dalian, China). The animals were individually weighed and divided into 8 groups of 5 animals. They were housed in standard cages at a room temperature of 24 ± 1 °C, humidity from 40% to 60%, and cycle lighting from 08:00 to 20:00. All mice had access to chow and water ad libitum.

Diet: All diets were purchased from Beijing Huafukang Bioscience Co., Ltd. After a one-week acclimatization period with a standard laboratory diet, the mice were fed for 8 weeks with a normal chow diet (4.3% fat) or high-fat diet (24% fat), with or without astaxanthin/*X. dendrorhous* (freeze-dried into powder, 7% fat, 200 μg/g astaxanthin). Astaxanthin (0.005% or 0.01%)/*X. dendrorhous* (10% or 20%, *w*/*w*) were added to the diet powder. The composition of the experimental diets is shown in Supplementary Table S1. The groups are shown in Table 3. 

### 4.4. Sample Collection

Body weight and food intake were recorded every week during the experimental period, and feces were collected at 4 weeks and 8 weeks for analysis of the microbial community. At the end of 8 weeks, the mice were fasted overnight before being anesthetized with ethyl ether. Eyeballs were extracted for collecting blood. Plasma samples were separated by centrifugation of blood at 4000 rpm for 10 min at 4 °C and then stored at −80 °C until analysis. Adipose tissue (epididymal, retroperitoneal, mesenteric, omental, and subcutaneous adipose) and organs (liver, kidney, spleen, and heart) were quickly removed and weighed, and the liver was stored at −80 °C until analysis. 

Liver tissue was accurately weighed, and anhydrous ethanol was added according to the ratio of weight (g):volume (mL) = 1:9. The mixture was homogenized in an ice water bath, centrifuged at 2500 rpm for 10 min, and the supernatant was taken to test the liver triglycerides and cholesterol.

### 4.5. Assays of Biochemical Parameters

The energy intake was calculated by the food intake × 4.73 kcal/g for the HFD groups and the food intake × 3.50 kcal/g for the ND groups.

The fat index was calculated by adipose tissue weight/body weight × 100%.

The plasma and liver concentrations of triglycerides and cholesterol were determined by the triglyceride assay kit and total cholesterol assay kit (Nanjing Jiancheng Bioengineering Institute, Nanjing, China), respectively.

### 4.6. Gut Microbiota Analysis

The collected feces were sent to Shanghai Majorbio Bio-pharm Technology Co., Ltd. for analysis of the microbial community using 16S amplicon sequencing. The sequencing method and the bioinformatics analysis were followed by Liu’s work [32]. 

### 4.7. Statistical Analyses

The results are expressed as the means ± SD (standard deviation of the mean), and analyses were based on one-way ANOVA, followed by the Fisher PLSD post hoc test if differences were significant. All statistical analyses were performed using SPSS 13.0 statistical software (SPSS Inc., Chicago, IL, USA), and the limit of statistical significance was set at *p* < 0.05.

## 5. Conclusions

In this study, (3R,3′R)-astaxanthin from *X. dendrorhous* and *X. dendrorhous* powder was added to the feed of the mice to investigate the effects of astaxanthin on the body weight, lipid metabolism, and gut microbiota. The results showed that astaxanthin and *X. dendrorhous* powder could prevent weight gain and elevated blood lipids mediated by a high-fat diet. In addition, astaxanthin and *X. dendrorhous* powder regulated gut microbiota, increased the abundance of beneficial bacteria, and maintained a healthier microflora structure, thus reducing the incidence of obesity.

## Figures and Tables

**Table 1:** Effects of astaxanthin on the physiological indexes of mice fed with a high-fat diet.

Physiological Indexes	HFD	HFD_ASX	HFD_2ASX	ND
Weight gain (g)	16.90 ± 2.65 ^a^	15.58 ± 1.95 ^a^	13.06 ± 2.10 ^b^	14.09 ± 3.66 ^a,b^
Energy intake (kcal/group/week)	426.37 ± 60.92 ^a^	379.81 ± 47.53 ^a,b^	444.31 ± 118.35 ^a^	321.72 ± 47.35 ^b^
Fat index%	11.48 ± 1.70 ^a^	5.91 ± 2.35 ^b,c^	4.39 ± 0.64 ^b^	6.69 ± 1.12 ^c^
Plasma triglyceride (mmol/L)	0.76 ± 0.28 ^a^	0.48 ± 0.12 ^b^	0.39 ± 0.14 ^b^	0.52 ± 0.14 ^b^
Plasma cholesterol (mmol/L)	5.21 ± 1.01 ^a^	5.28 ± 1.17 ^a^	3.86 ± 1.21 ^b^	3.82 ± 0.54 ^b^
Liver triglyceride (μmol/g)	33.13 ± 7.82 ^a^	29.81 ± 5.53 ^a^	24.45 ± 6.10 ^b^	24.60 ± 4.55 ^b^
Liver cholesterol (μmol/g)	18.20 ± 2.30 ^a^	13.72 ± 2.70 ^b^	13.31 ± 1.62 ^b^	13.39 ± 2.09 ^b^

^a^, ^b^, ^c^ Different letters indicate statistically significant variations between groups (*p* < 0.05). HFD: High-fat diet; HFD_ASX: High-fat diet + astaxanthin (0.005%); HFD_2ASX: High-fat diet + astaxanthin (0.01%); ND: Normal diet.

**Table 2:** Effects of *X. dendrorhous* powder on the physiological indexes of mice fed with a high-fat diet.

Physiological Indexes	HFD’	HFD’_XD	HFD’_2XD	ND’
Weight gain (g)	18.08 ± 3.08 ^a^	17.26 ± 2.95 ^a^	13.63 ± 2.70 ^b^	14.47 ± 2.85 ^b^
Energy intake (kcal/group/week)	419.36 ± 46.08 ^a^	413.13 ± 72.88 ^a^	408.23 ± 87.34 ^a^	354.62 ± 55.41 ^b^
Fat index%	6.97 ± 1.87 ^a^	3.58 ± 0.67 ^b^	3.28 ± 0.86 ^b^	4.21 ± 1.46 ^b^
Plasma triglyceride (mmol/L)	1.06 ± 0.24 ^a^	0.48 ± 0.10 ^b^	0.59 ± 0.13 ^b^	0.56 ± 0.10 ^b^
Plasma cholesterol (mmol/L)	5.22 ± 0.73 ^a^	3.86 ± 1.59 ^b^	4.69 ± 0.29 ^b^	4.39 ± 0.67 ^b^
Liver triglyceride (μmol/g)	28.26 ± 2.43 ^a^	23.38 ± 2.95 ^b^	22.20 ± 1.56 ^b^	25.33 ± 5.80 ^b^
Liver cholesterol (μmol/g)	19.02 ± 2.1 ^a^	12.63 ± 1.0 ^b^	11.90 ± 2.0 ^b^	9.54 ± 0.63 ^c^

^a, b, c^ Different letters indicate statistically significant variations between groups (*p* < 0.05). HFD’: High-fat diet; HFD’_XD: High-fat diet + *X. dendrorhous* (10% *w*/*w*); HFD’_2XD: High-fat diet + *X. dendrorhous* (20% *w*/*w*); ND’: Normal diet.

**Table 3:** Experimental groups.

Group	Ingredient
HFD	High-fat diet (Base diet + 24% fat)
HFD_ASX	High-fat diet + astaxanthin (0.005%)
HFD_2ASX	High-fat diet + astaxanthin (0.01%)
ND	Normal diet
HFD’	High-fat diet (Base diet + 24% fat)
HFD’_XD	High-fat diet + *X. dendrorhous* (10% *w*/*w*)
HFD’_2XD	High-fat diet + *X. dendrorhous* (20% *w*/*w*)
ND’	Normal diet

## Supplementary Materials

The following are available online at https://www.mdpi.com/1660-3397/17/6/337/s1, Figure S1: Effects of astaxanthin on gut microbiota at the genus level of the mice fed with a high-fat diet, Figure S2: Effects of *X. dendrorhous* powder on gut microbiota at the genus level of the mice fed with a high-fat diet, Table S1: The composition of the experimental diets.
