# Supplementary material for: Xanthophyllomyces dendrorhous-Derived Astaxanthin Regulates Lipid Metabolism and Gut Microbiota in Obese Mice Induced by A High-Fat Diet
Source: Mar Drugs. 2019 Jun 5;17(6):337. doi: 10.3390/md17060337 (PMC6627754; doi:10.3390/md17060337)
Supplement: Supplementary file 1 [file marinedrugs-17-00337-s001.pdf]

marine drugs

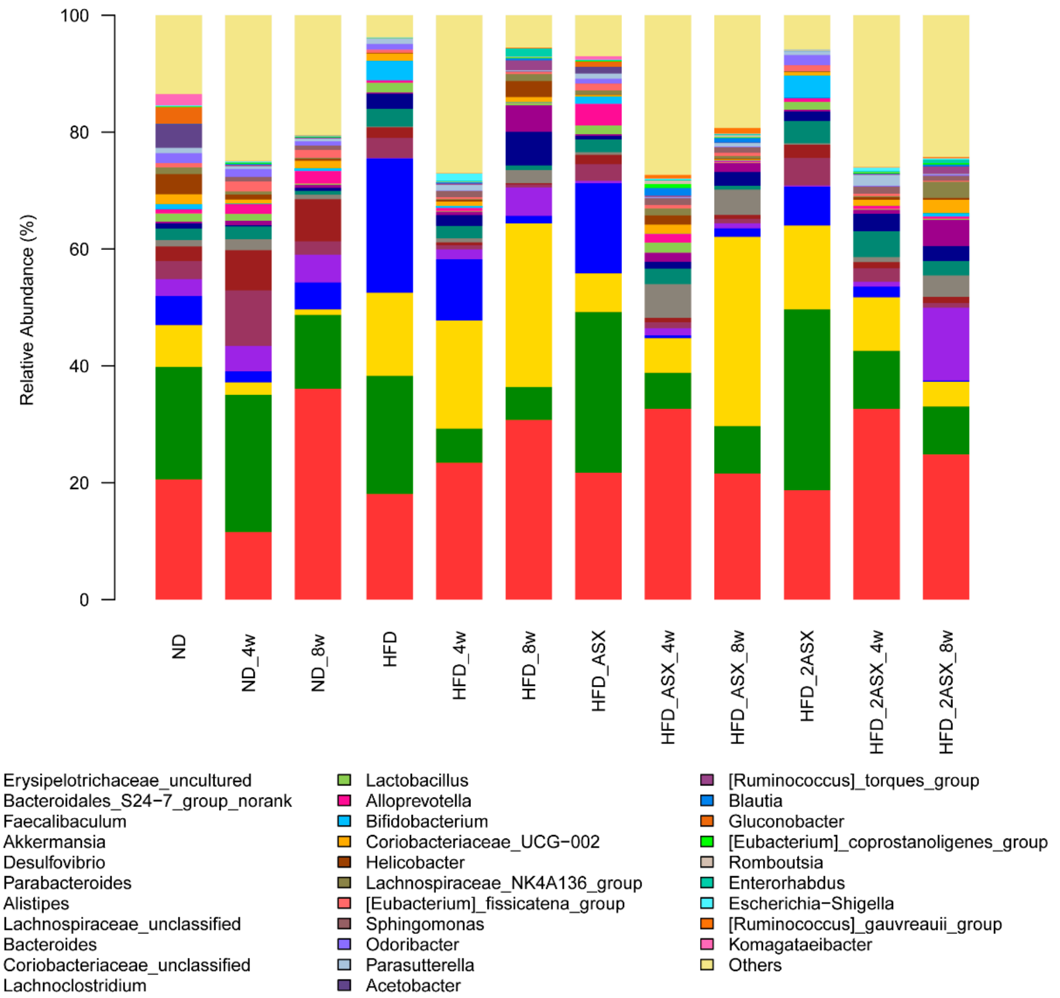

Figure S1: Effects of astaxanthin on gut microbiota at the genus level of the mice fed with a high-fat diet

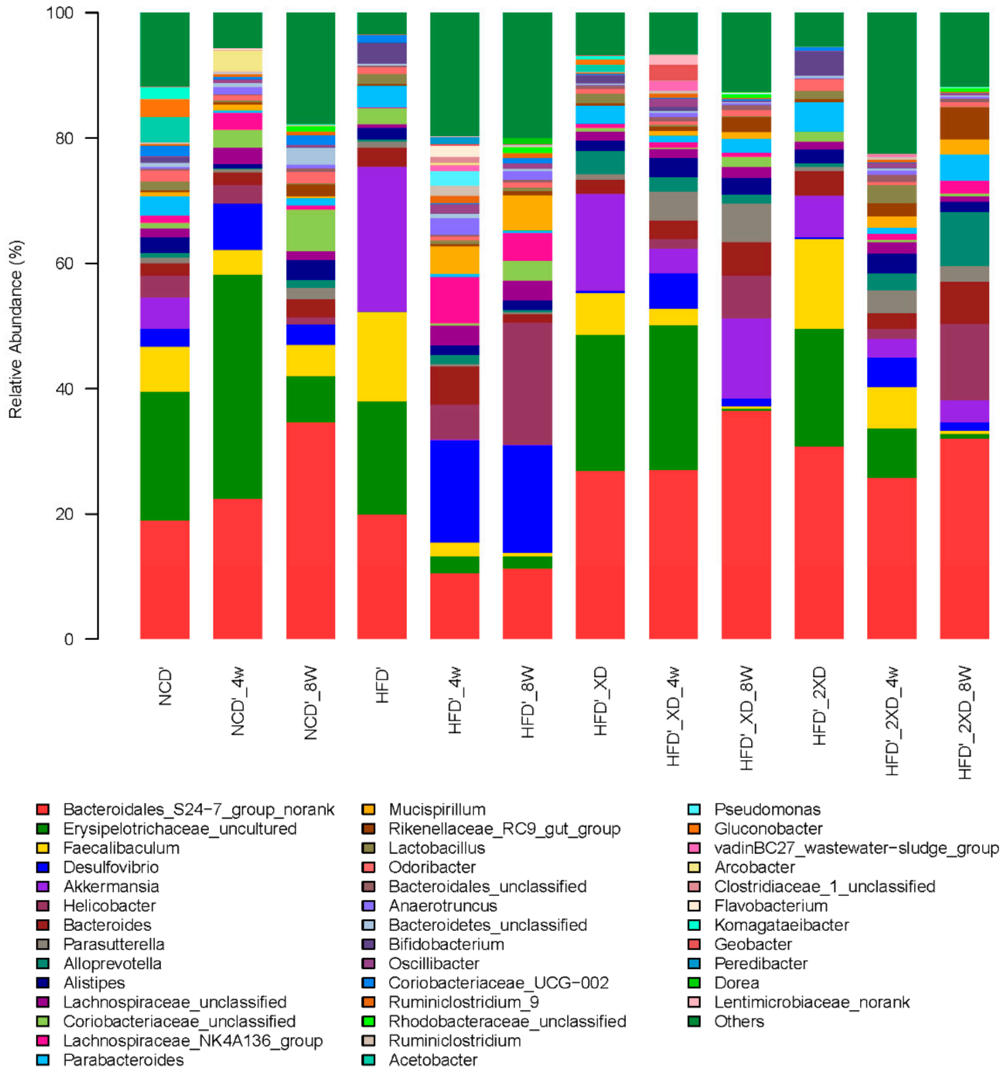

Figure S2: Effects of *X. dendrorhous* powder on gut microbiota at the genus level of the mice fed with a high-fat diet

Table S1: The composition of the experimental diets.

ingredient	NCD/g	HFD/g
casein	189.58	233.06
L-cystine	2.84	3.5
cornstarch	298.59	84.83
maltodextrin	33.18	116.53
sucrose	331.77	201.36
cellulose	47.4	58.26
soybean oil	23.7	29.13
lard	18.96	206.84
M1002 mineral mix	9.48	11.65
calcium hydrogenphosphate	12.32	15.15
calcium carbonate	5.21	6.41
patassium citrate	15.64	19.23
V1001 vitamin mixture	9.48	11.56
choline bitartrate	1.9	2.33
food dye	0.047	0.058
total	1000	1000
